# Neglected Australian Arboviruses Associated With Undifferentiated Febrile Illnesses

**DOI:** 10.3389/fmicb.2019.02818

**Published:** 2019-12-06

**Authors:** Narayan Gyawali, Andrew W. Taylor-Robinson, Richard S. Bradbury, Wayne Pederick, Helen M. Faddy, John G. Aaskov

**Affiliations:** ^1^Institute of Health and Biomedical Innovation, Queensland University of Technology, Brisbane, QLD, Australia; ^2^School of Health, Medical and Applied Sciences, Central Queensland University, Rockhampton, QLD, Australia; ^3^Mosquito Control Laboratory, QIMR Berghofer Medical Research Institute, Brisbane, QLD, Australia; ^4^School of Health, Medical and Applied Sciences, Central Queensland University, Brisbane, QLD, Australia; ^5^Research and Development, Australian Red Cross Blood Service, Brisbane, QLD, Australia

**Keywords:** alphavirus, arbovirus, Australia, flavivirus, prevalence, Queensland, Ross River virus, undifferentiated febrile illness

## Abstract

Infections with commonly occurring Australian *ar*thropod-*bo*rne arboviruses such as Ross River virus (RRV) and Barmah Forest virus (BFV) are diagnosed routinely by pathology laboratories in Australia. Others, such as Murray Valley encephalitis (MVEV) and Kunjin (KUNV) virus infections may be diagnosed by specialist reference laboratories. Although Alfuy (ALFV), Edge Hill (EHV), Kokobera (KOKV), Sindbis (SINV), and Stratford (STRV) viruses are known to infect humans in Australia, all are considered ‘neglected.’ The aetiologies of approximately half of all cases of undifferentiated febrile illnesses (UFI) in Australia are unknown and it is possible that some of these are caused by the neglected arboviruses. The aims of this study were to determine the seroprevalence of antibodies against several neglected Australian arboviruses among residents of Queensland, north-east Australia, and to ascertain whether any are associated with UFI. One hundred age- and sex-stratified human plasma samples from blood donors in Queensland were tested to determine the prevalence of neutralising antibodies against ALFV, BFV, EHV, KOKV, KUNV, MVEV, RRV, SINV, and STRV. The seroconversion rates for RRV and BFV infections were 1.3 and 0.3% per annum, respectively. The prevalence of antibodies against ALFV was too low to enable estimates of annual infection rates to be determined, but the values obtained for other neglected viruses, EHV (0.1%), KOKV (0.05%), and STRV (0.05%), indicated that the numbers of clinical infections occurring with these agents are likely to be extremely small. This was borne out by the observation that only 5.7% of a panel of 492 acute phase sera from UFI patients contained IgM against any of these arboviruses, as detected by an indirect immunofluorescence assay. While none of these neglected arboviruses appear to be a cause of a significant number of UFIs in Australia at this time, each has the potential to emerge as a significant human pathogen if there are changes to their ecological niches.

## Introduction

Arboviruses, defined as viruses that replicate in both vertebrate hosts and invertebrate vectors and which are transmitted between vertebrate hosts by biting arthropods (such as mosquitoes, ticks, sandflies, and midges), present a significant public health risk in Australia and internationally (Wilder-Smith et al., 2017). The recent epidemics and intercontinental spread of hitherto obscure diseases such as chikungunya and Zika have highlighted the significant threat to global health security presented by emerging arthropod-borne viruses (Gyawali et al., 2016). More than 75 arboviruses have been identified in Australia (Centers for Disease Control and Prevention, 2019), and while only a few of these are known to cause disease in humans there are limited or no data regarding the potential pathogenicity for humans of most others (Gyawali et al., 2017b).

Although most infections caused by arboviruses are mild or asymptomatic, those known to cause disease in humans in Australia include Ross River (RRV) and Barmah Forest (BFV), alphaviruses that elicit a debilitating and sometimes chronic polyarthritis (Fraser, 1986; Phillips et al., 1990). The flaviviruses Murray Valley encephalitis (MVEV) and West Nile virus (Kunjin strain, KUNV) cause encephalitis, while dengue viruses (DENV) cause a febrile illness and, less commonly, haemorrhagic fever (French, 1952; Halstead et al., 1970; Muller et al., 1986).

For almost a decade after the identification of RRV (Doherty et al., 1963), only small numbers of patients were diagnosed with a clinical infection with this agent because diagnostic testing was available only in a research setting and using an in-house methodology. Following the development and commercial release of an enzyme-linked immunosorbent assay (ELISA) to detect anti-RRV immunoglobulin IgM (Oseni et al., 1983), the number of patients diagnosed annually rose from 50 to 200 during the 1980s and 4,000 to 9,000 in the present decade. A similar but smaller increase in the number of cases of BFV disease was observed when a commercial diagnostic assay for that infection became available (Australian Government Department of Health, 2019). Moreover, epidemic polyarthritis, the disease caused by RRV infection, was made a nationally notifiable disease in 1991 (Hargreaves et al., 1995). Clinical infections with the viruses Edge Hill (EHV), Kokobera (KOKV), KUNV, and MVEV can be confirmed in some reference laboratories but only tests for suspected KUNV and MVEV are performed in these specialised facilities on a routine basis (Australian Government Department of Health, 2019).

Other Australian arboviruses, such as Alfuy (ALFV), Sindbis (SINV), and Stratford (STRV), have been associated with human disease (Doherty et al., 1969; Doherty, 1973; Hawkes et al., 1985, 1993; Boughton et al., 1986; Aaskov et al., 1993). However, each is thought to cause mild symptoms and no outbreaks of disease due to any of these have been reported in Australia (Australian Government Department of Health, 2019). In the absence of routine testing by all diagnostic laboratories for infection with these remaining neglected alpha- and flaviviruses, it remains possible that some patients with undifferentiated febrile illnesses (UFIs) – fever without diagnosed cause – are experiencing an infection with one of these ‘neglected’ viruses (Gyawali et al., 2017a). A retrospective study in Western Australia, undertaken from July 2000 to July 2003, identified 3,218 UFI cases (Ingarfield et al., 2007). Another investigation, from July 2008 to June 2011, conducted in a hospital in Far North Queensland found that 58.8% of patients with febrile illnesses received no definitive diagnosis (Susilawati and McBride, 2014).

Many UFIs go undiagnosed because the aetiological agent is novel or not known to cause human disease or because the tests to diagnose the infection are not available or are not sufficiently sensitive to be of clinical utility. The funding model for diagnostic pathology in Australia restricts the likelihood that a patient’s treating physician would request tests for infection with a little-known arbovirus even if they have considered one in their differential diagnosis (Gyawali et al., 2016). This study aimed to determine rates of infection among adult residents of Queensland with neglected alphaviruses and flaviviruses – and to determine whether any of these are associated with UFIs.

## Materials and Methods

### Study Design, Samples, and Research Setting

Two sets of samples were collected for this study. For the first, 100 de-identified plasma samples (3–5 mL) from 50 female and from 50 male, age-stratified, adult (20–69 years) Australian blood donors were provided by the Australian Red Cross Blood Service (approved by the Australian Red Cross Blood Service Human Research Ethics Committee, approval no. 12-03QLD-10). The samples were collected in 2015 throughout the state of Queensland. The prevalence of anti-RRV IgG in subjects from northern Australia observed by Aaskov et al. (1981) was 28/62 (45.1%). If it is assumed that the infection rate with neglected arboviruses is the same as that of RRV infections, the minimum sample size (for blood donor samples) required to detect the prevalence of such arboviral infections with 95% confidence at 0.1 margin of error would be 96. Hence, a sample collection of 100 was considered to provide statistically valid data.

The second sample set consisted of 492 de-identified sera from UFI patients, collected during January 2015 and December 2016 (during the Queensland wet season) by QML Pathology (Central Queensland University Human Research Ethics Committee approval no. H15/03-041). Inclusion criteria were that samples were submitted to QML Pathology from febrile patients for a variety of laboratory tests, including arbovirus serology, and with clinical notes such as ‘fever,’ ‘fever of unknown origin,’ ‘pyrexia of unknown origin,’ ‘viral studies,’ or ‘undifferentiated fever/febrile illness.’ Samples from patients with a laboratory diagnosis of an autoimmune or neoplastic condition, or of an infection with a bacterial, parasitic or viral agent – including RRV, BFV, and DENV – were excluded.

### Determination of Background Infection Rates With Australian Alphaviruses and Flaviviruses Among Adults in Queensland

Neutralisation tests with plasma from blood donors employed the following prototype strains of viruses: ALFV MRM3929 (Whitehead et al., 1968), BFV BH2193 (Marshall et al., 1982), EHV C281, KOKV CH112820, KUNV MRM16, RRV T48, SINV MRM 39, STRV C338 (Doherty et al., 1963), and MVEV MVE/1/1951 (French, 1952). Australian alphaviruses and flaviviruses were reported to produce plaques on monolayers of porcine stable equine kidney cells (PS-EK; Gorman et al., 1975) but BFV did not do so in our experience. As all alphaviruses of interest, including BFV, formed plaques on baby hamster kidney (BHK-21) cells, the study employed PS-EK cells for neutralisation tests involving flaviviruses and BHK cells for tests involving alphaviruses.

Two hundred μL of plasma was inactivated by heating at 56°C for 30 min, then diluted 1 in 10 in serum-free Roswell Park Memorial Institute 1640 (RPMI-1640) medium (Sigma-Aldrich, St. Louis, MO, United States). This solution was mixed with an equal volume of virus stock diluted to contain between 50 and 60 plaque forming units (pfu) and added to cell monolayers at 37°C for 2 h to enable non-neutralised virus to adsorb to cells. Two mL of 0.75% w/v carboxymethyl cellulose (CMC, Sigma-Aldrich)/RPMI 1640 was then added and the plates were incubated at 37°C in an atmosphere of 5% v/v CO_2_/air for an additional 2–4 days, depending on the virus being employed, i.e., 2 days for alphaviruses and 4 days for flaviviruses. The CMC medium was then removed and the cell monolayers were fixed and stained with 0.05% w/v crystal violet (Sigma-Aldrich) in formaldehyde (1% v/v) and methanol (1% v/v). Plasma samples that reduced virus plaque numbers by ≥ 50% were recorded as reactive.

**FIGURE 1:**
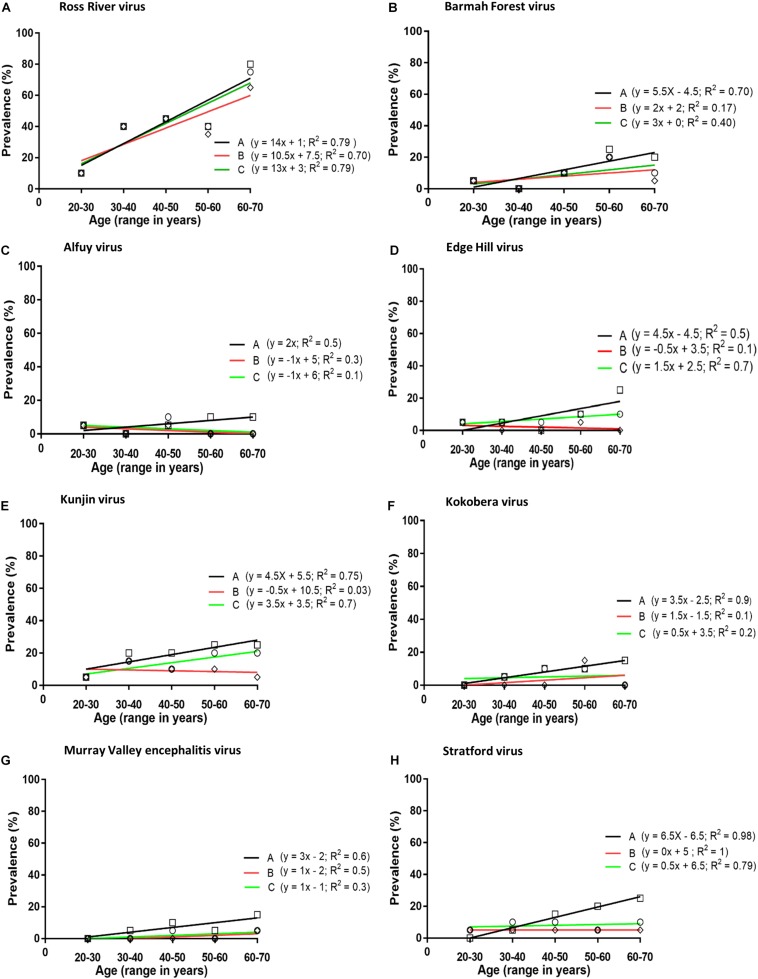
Prevalence of neutralising antibodies against arboviruses **(A–H)** in plasma obtained from blood donors. Analysis performed by plotting prevalence of neutralising antibodies against age groups: Line A (squares), neutralising antibodies against any virus considered to represent an infection with that virus; i.e., ignoring the possibility of serological cross-reactions; Line B (diamonds), including samples that neutralised only one virus; i.e., excluding all samples which may have contained cross-reactive antibodies; Line C (circles), each subject was infected with only a single virus and it was the one against which the highest neutralising activity was observed; i.e., all other neutralising activity was assumed to be due to cross-reactive antibodies.

The prevalence of antibodies against each virus was plotted against the age of the donors and the line of best fit was determined by linear regression (Figure 1). The annual infection rate was calculated from the slope of the line of best fit. Given previous reports of cross-reactivity between flaviviruses by polyclonal antisera (De Madrid and Porterfield, 1974; Calisher et al., 1989; Hall et al., 1990), and of mouse monoclonal antibodies that recognise epitopes on the E1 or E2 proteins of multiple alphaviruses, e.g., 2A2C3 (Roehrig, 1986), it is possible that plasma samples that recognised more than one alphavirus or more than one flavivirus might represent serological cross-reactivity, rather than be due to infections with multiple viruses. Therefore, the results shown in Tables 1, 2 were analysed based on three different assumptions: (1) The presence of neutralising antibody against any virus represented an infection with that virus; i.e., ignoring the possibility of serological cross-reactions (Figure 1, line A); (2) Analyses were performed with samples that neutralised only one virus; i.e., excluding all samples that may have contained cross-reactive antibodies (Figure 1, line B); and (3) Each subject was infected with only a single virus and it was the one against which the highest neutralising activity was observed; i.e., all other neutralising activity was due to cross-reacting antibodies (Figure 1, line C).

**TABLE 1:** Plasma samples that neutralised one or more Australian flaviviruses by fifty *per cent* or more (*n* = 100).

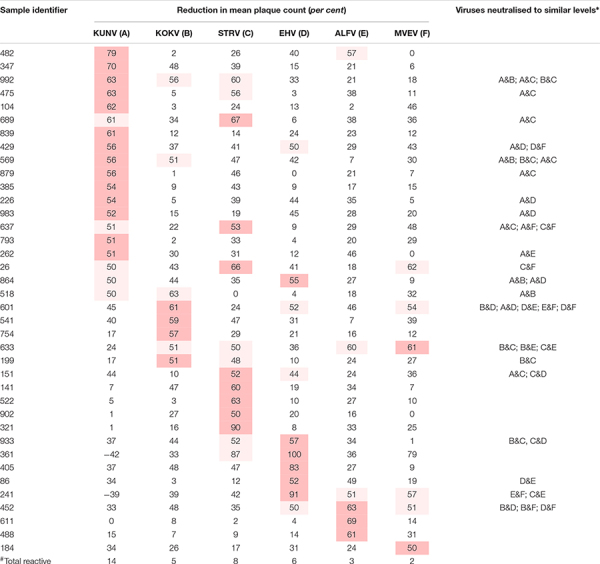

*Values for reactive samples are shaded, with the darker shade notifying the most reactive. ^∗^There was no significant difference (*p* > 0.05, Student’s *t*-test) between the neutralisation of the virus pairs indicated. ^#^Numbers are based on an assumption that the subject had been infected with a single virus and it was the one against which the highest neutralising activity (darker shade) was observed.*

**TABLE 2:** Plasma samples that neutralised one or more Australian alphaviruses by fifty *per cent* or more (*n* = 100).

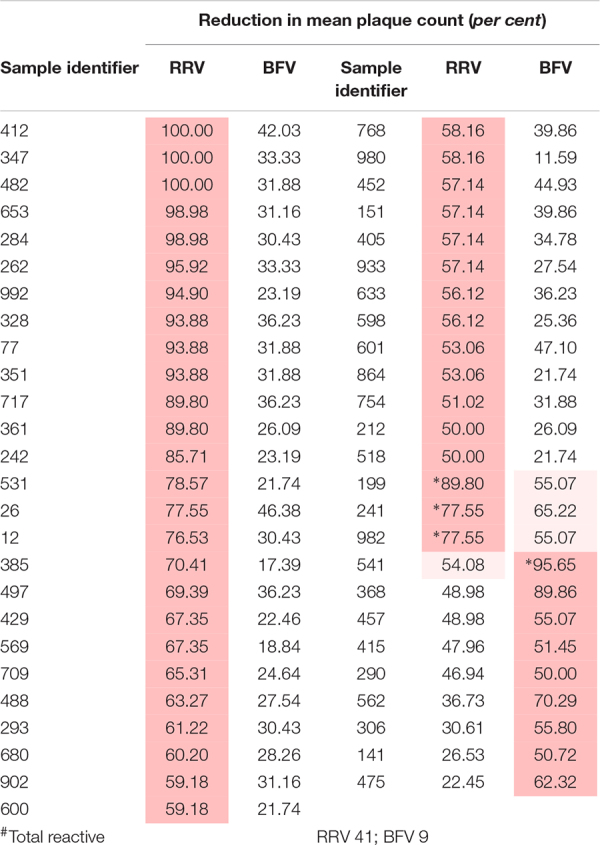

*^#^The numbers are based on an assumption that the subject had been infected with a single virus and it was the one against which the highest neutralising activity (darker shade) was observed. ^∗^The value is significantly greater (*p* < 0.05, Student’s *t*-test) than the corresponding value in the same row.*

### Detection of IgM Antibodies Against Australian Alphaviruses and Flaviviruses in Acute Phase Sera From UFI Patients in Queensland

A modification of the indirect immunofluorescence assay (IFA) of Aaskov and Davies (1979) was employed to detect IgM antibodies against a range of alphaviruses (BFV, RRV, and SINV) and flaviviruses (ALFV, EHV, KOKV, KUNV, MVEV, and STRV) in sera from UFI patients. Briefly, 40 μL of a suspension of virus-infected C6/36 cells (approximately 1.25 × 10^4^ cells) was aliquoted onto each well of a 12-well Teflon-coated IFA slide (Cell Line Diagnostic, Thermo Scientific, Dreieich, Germany). After allowing cells to settle, the liquid in each well was aspirated and cells were air-dried (at room temperature for 15 min) before being fixed in ice-cold acetone (2 min). Fifty μL of serum from each UFI patient was diluted 1 in 20 in phosphate-buffered saline (PBS), pH 7.2, and added to each spot on the IFA slide containing infected cells before the slide was incubated at room temperature for 45 min. Slides were then washed in PBS, three times for 10 min each. Secondary antibody [FITC-labelled polyclonal rabbit anti-human IgM (Dako, Glostrup, Denmark)], was added to the cells and incubated at room temperature for 30–45 min before undergoing three further washes in PBS. A glass cover slip was placed over the slide and cells were viewed under a UV microscope (Eclipse, Nikon, Minato, Japan) using phloem illumination. Cells were considered to be infected if a clear green fluorescent signal was observed in the cytoplasm.

In order to exclude the possibility that the apparent anti-viral IgM reactions in IFAs were due to IgM rheumatoid factor (RF) attaching to anti-viral IgG, all IgM-reactive samples were tested for the presence of RF using a latex agglutination assay, performed according to the manufacturer’s instructions (Dutch Diagnostics, Zutphen, Netherlands). Briefly, 50 μL of the UFI sample and one drop of RF-latex reagent were mixed with a stirrer and then shaken at 100 rpm for 2 min. Semi-quantitative assays were performed by making twofold dilutions of serum in normal saline before being added to the RF-latex reagent. The approximate concentration of RF was calculated by multiplying the RF titre by 8 IU/mL, as suggested by the manufacturer.

All IgM-reactive sera were then tested for the presence of neutralising antibodies against the viruses recognised by IgM antibody in the IFA assay, by the method described above.

## Results

### Prevalence of Neutralising Antibodies to Australian Alphavirues and Flaviviruses in Blood Donors

Plasma samples from 63 of the panel of 100 donors neutralised one or more of the arboviruses against which they were tested. Thirty-eight samples neutralised one or more flavivirus (Table 1), and for 21 of these reactive samples no single virus was neutralised to a greater extent than all others (*p* > 0.05; Student’s *t*-test). Samples 361 and 241 consistently enhanced the number of plaques formed by KUNV.

Similarly, 51 of the 100 samples neutralised one or more alphaviruses (Table 2). Of these, samples from 39 donors neutralised RRV and eight neutralised BFV to a greater extent than another; i.e., there was a significant difference between the reductions in plaque count (*p* < 0.05). Only four samples neutralised both RRV and BFV.

The prevalence of antibody to a virus increased with the age of the donor, except for ALFV, but only with RRV, EHV, and KUNV was there a significant linear association between antibody prevalence and age (*p* < 0.05). If it is assumed that each subject was infected with only a single virus and it was the one against which the highest neutralising activity was observed (assumption number 3, section ‘Materials and Methods’), the annual infection rate calculated for viruses among blood donors in Queensland were: RRV 1.3%, BFV 0.3%, EHV 0.15%, KUNV 0.15%, MVEV 0.1%, and STRV 0.05%.

### Prevalence of IgM Antibodies to Australian Alphavirues and Flaviviruses in Acute Phase Sera From UFI Patients

Four-hundred and ninety-two acute phase sera were obtained from patients between 12 and 75 years of age (median of 45 years); 238 (48.4%) were male and 254 (51.6%) were female. Thirty-two samples contained IgM antibody that reacted with C6/36 cells infected by either an alphavirus or a flavivirus, or by several viruses. As the extent of cross-reactivity observed in these assays to detect anti-arboviral IgM was unexpected (Calisher et al., 1989), neutralisation tests were performed with each sample against all of the viruses to which IgM appeared to be reacting. The four samples (35, 91, 440, and 456 in Table 3) that failed to neutralise any of the arboviruses, by ≥ 50%, with which they reacted in IFA were regarded as non-reactive and excluded from subsequent analyses. Sample 456 consistently enhanced the number of plaques formed by incubation with KUNV.

**TABLE 3:** Recognition of Australian alphaviruses and flaviviruses by IgM antibody in sera from UFI patients in indirect immunofluorescence assays.

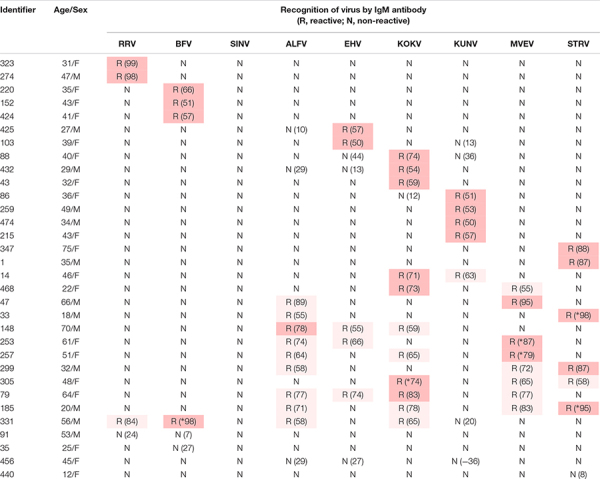

*Values for reactive samples are shaded, with the darker shade notifying the most reactive. The neutralisation (per cent) of viruses recognised by IgM antibodies, identified by the patient serum, is shown in parenthesis. ^∗^Neutralisation value is significantly greater (*p* < 0.05, Student’s *t*-test) than any other values within the same row (i.e., for the same serum sample).*

Twenty-eight of the 32 IgM-reactive samples (5.7% of 492) neutralised one or more alphavirus or flavivirus (Table 3). Of these, 16 samples (3.2% of 492) neutralised a single virus (2 RRV, 3 BFV, 4 KUNV, 3 KOKV, 2 EHV, and 2 STRV). Twelve IgM-reactive samples neutralised more than one virus with one (331 in Table 3) neutralising both alphaviruses and flaviviruses. Only two of the 28 (7.1%) arbovirus-neutralising sera contained RF (274,64 IU/mL; 331,1104 IU/mL).

Estimates of numbers of clinical infections with these viruses were made using a range of total infection to clinical (symptomatic) infection ratios and the seroprevalence data employing assumptions 3, section ‘Materials and Methods,’ above (Table 4).

**TABLE 4:** Estimates of subclinical and clinical infection rates with Australian arboviruses in the Queensland population using the annual infection rates determined from this study.

**Virus**	**^∗^Annual infection rate per cent per annum**	**Estimated annual number of infections**	**Assumed clinical infection rate (clinical/total infection rate)**	**Clinical infections estimated annually**	**Average annual number of cases reported**
RRV	1.3	63,960	20%	12,792	2,779
			10%	6,396	
			5%	3,195	
			1%	639	
BFV	0.3	14,760	20%	2,952	857
			10%	1,476	
			5%	723	
			1%	148	
ALFV	0.2	9,840	20%	1,968	NA
			10%	984	
			5%	492	
			1%	98	
EHV	0.15	7,380	20%	1,478	NA
			10%	784	
			5%	362	
			1%	73	
KOKV	0.05	2,460	20%	492	NA
			10%	246	
			5%	123	
			1%	24	
KUNV	0.3	14,760	20%	2,952	4
			10%	1,476	
			5%	738	
			1%	148	
MVEV	0.10	4,920	20%	984	1
			10%	492	
			5%	246	
			1%	49	
STRV	0.05	2,460	20%	492	NA
			10%	246	
			5%	123	
			1%	24	

*2017 census of Queensland estimated 4.92 million population. ^∗^The values presented are the annual infection rate per annum for each virus determined in this study. NA, data not available.*

## Discussion

This study suggests that only a small proportion of UFIs in Australia are caused by flaviviruses for which virological and serological diagnostic testing is not performed on a routine basis. The low clinical infection rate is supported by the low rate of sub-clinical or inapparent infection with these viruses among blood donors.

The annual RRV infection rate determined by our investigation (1.3%) is in close agreement with that observed by Doherty et al. (1966) (1.4 to 3.2% for residents of Cape York Peninsula, North Queensland), by Doherty et al. (1968) (1.5% for residents of Innisfail, Far North Queensland) and by Aaskov et al. (1981) (1.4% for residents of Central and North Queensland). Taken together, these results suggest that there have been constant and continuous RRV infections in Queensland for decades. Furthermore, these findings also indicate that there was no major bias in the sampling of sera used in the current study.

The annual rate of infections with BFV (0.3%) and its seroprevalence (9.0%) were similar to those reported previously for Queensland residents [0.23% prevalence rate and 6.5% seroprevalence (Phillips et al., 1990)]. However, the seroprevalence was significantly higher (Chi–square test, *p* < 0.05) than that reported previously in Australia by Faddy et al. (2015) among blood donors in Queensland (2.9%), by Vale et al. (1986) among blood donors on the south coast of New South Wales (2.9%), by Hawkes et al. (1987) in inland (0.3%) and coastal regions (6%) of New South Wales. Some of these differences may reflect the sensitivities and specificities of the various assays employed; e.g., the neutralisation test employed in the current study and a commercial ELISA used by Faddy et al. (2015), or the type of sample collection and hypothesis of the particular study. While use of separate assay methodologies may have influenced the differences in seroprevalence values observed between Queensland and other states, it is also possible that both the abundance and species of mosquito vectors at geographically distant localities played a role (Webb et al., 2016).

The prevalence of antibodies against several neglected flaviviruses suggested they are associated with human infection. This conclusion is broadly in line with prior reports from Queensland (Doherty, 1973), New South Wales (Hawkes et al., 1985, 1993), and Victoria (Williams et al., 2013). In the present investigation annual seroconversion rates for flaviviruses (EHV 0.15%, KOKV 0.05%, MVEV 0.10%, and STRV 0.05%) (Figures 1A–E) were significantly lower than those that have been determined for the alphaviruses RRV and BFV. However, an annual seroconversion rate for KUNV of 0.3% is similar to that for BFV. This overall disparity between rates of infection with alphaviruses and flaviviruses might reflect differences in their transmission cycles, particularly the vectors and reservoirs that are involved.

Kunjin shares a number of epidemiological characteristics with MVEV, including reservoir hosts (wading birds, particularly the rufous night heron; *Nycticorax caledonicus*) and mosquito vector species (particularly the common banded mosquito; *Culex annulirostris*) (Evans et al., 2009; Prow, 2013). However, in this study, antibodies against KUNV were more prevalent than those against MVEV among, predominantly, urban dwelling blood donors. This is in contrast to the observations of Doherty et al. (1959) in Far North Queensland, where approximately 50% of the residents of one aboriginal community had antibodies to MVEV but only 8% possessed anti-KUNV antibodies. Lower rates of infection with MVEV compared to those reported by Doherty et al. (1959) may reflect the transitory presence of the putative hosts of MVEV in nature, migratory water birds (Kingsford and Thomas, 1995). It may also reflect greater transmission of MVEV at the more northern latitude of Doherty’s study than that which occurs elsewhere in the state of Queensland, from where the samples for this study were acquired. The most northerly part of Queensland from which samples were collected for this study was Cairns, which is 500 km south of Doherty’s study site. In Western Australia also, MVEV transmission is more common in the northernmost Kimberley region than in the more southern central Pilbara region, where KUNV is most prevalent (Prow, 2013). Moreover, both annual and longer-term alterations in rainfall and climate patterns in the 60 years since the publication of Doherty’s data may have altered the transmission rates of KUNV and MVEV in Queensland (Prow, 2013). Further investigation is required to substantiate our finding of a potential emergence of KUNV in north-east Australia. The number of sera containing anti-ALFV antibodies were too small to detect an increase in the prevalence of anti-ALFV antibodies with increasing age of the blood donors, even if it was occurring, suggesting that ALFV infections may occur sporadically rather than on a regular basis.

Confounders of the observed seroprevalence of anti-flavivirus antibodies were the failure to test for anti-DENV antibodies and the possibility that blood donors had been vaccinated against either yellow fever or Japanese encephalitis. However, neither yellow fever nor Japanese encephalitis is endemic to Australia so immunisation would be recommended only in the event of international travel. Use of these vaccines among the Australian population, especially in regional communities, is assumed to be very low and this is unlikely to have influenced the results obtained. There are both locally acquired DENV infections and large numbers of Australian tourists returning with DENV infection acquired overseas. However, few, if any, of the levels of anti-flavivirus neutralising activity detected among plasma from blood donors could have been attributed to cross-reactive anti-DENV antibody from healthy blood donors.

For many of the viruses studied, the ratio of total infections to clinical infections in humans is unknown. From estimates made with the data obtained in this study (Table 4), it appears that approximately 5% of infections with RRV and BFV in Queensland are clinical infections severe enough to require medical attention and so be reported. If it is assumed that only a small proportion, e.g., 1%, of infections caused by flaviviruses studied in the current study results in disease, there would be at least 416 clinical infections caused by these viruses in Queensland annually.

Approximately 5.7% of UFI subjects had been infected recently (i.e., this proportion of sera contained anti-viral IgM) by one of the neglected Australian flaviviruses for which a diagnostic test was not requested or is not available in commercial laboratories. However, as these samples were collected during the wet summer months when the rate of transmission of the common arboviruses RRV, BFV, and DENV is highest, the annual value might be less than 5.7%. The detection of anti-RRV and anti-BFV IgM antibody in sera from a small number of these patients was unexpected because serology tests to determine the infection caused by these viruses might have been requested and performed on these samples using commercial ELISA. However, the ethical constraints on this study prevented retrospective access to any related serology that may have been conducted on these samples by a pathology laboratory. The PanBio ELISA kits, the only commercial tests for diagnosis of RRV and BFV currently available in Australia, have a sensitivity of 98.5% for IgM detection (Harley et al., 2001).

The extent of IgM cross-reactivity between the flaviviruses also was unexpected, but is reflected in the number of ‘flavivirus-unspecified’ reports made to the Australian Government National Notifiable Disease Surveillance System (Australian Government Department of Health, 2019). However, by combining the IgM data with those for neutralisation tests (Table 3), it was possible to implicate a given virus as the likely causative agent of a recent UFI. In most instances in which cross-reactive IgM antibody was detected, neutralising antibodies could be identified against the same viruses. The absence of detectable RF in almost all samples suggested it was not responsible for false positive IgM reactions.

Enhancement of infection with KUNV was noted with some samples examined in this study. There is an extensive literature describing enhancement of flavivirus infection *in vitro* by cross-reactive and non-neutralising antibodies, starting with the report of Hawkes (1964), who also described enhancement of infection by West Nile virus (Hawkes, 1964). However, antibody-mediated enhancement of infection with KUNV, an Australian strain of West Nile virus, by human antisera has not been reported previously. It is not known if the PS-EK cells that were used in this study express the Fc receptors required for antibody-dependent enhancement of viral infection to occur (Kliks and Halstead, 1983) or if another mechanism was responsible for our observations.

While this study identified neglected arboviruses that may cause UFI, the prevalence of these infections may have been elevated by the samples tested coming from a collection for which arbovirus serology or ‘viral studies’ had been requested and while the presence of anti-viral IgM indicates a recent infection, it does not indicate that the infection has caused the clinical signs and symptoms observed. It should be noted that the aetiology of disease in at least 94% of the patients remained undetermined.

## Conclusion

The occurrence of UFI due to ‘neglected’ Australian flaviviruses like ALFV, EHV, KOKV, and STRV appears to be extremely low and the reported annual infection rates of these viruses suggest that this has always been the case. However, they do infect humans and so population growth, societal changes (such as increased urbanisation and travel) and variations in the ecosystem (climate-related or other) could have a profound effect on their transmission cycles, with consequent changes to the rates of human infection. Under these circumstances the current routine diagnostic testing regimes would not detect a rise in the rate of infection with ALFV, EHV, KOKV, and STRV. In order to monitor any future changes in the transmission of these viruses, there is a strong case to be made for the systematic testing of a sub-sample of UFI cases by state or national public health laboratories.

## Data Availability Statement

All datasets generated for this study are included in the article/supplementary material.

## Ethics Statement

The studies involving human participants were reviewed and approved by the Central Queensland University Human Research Ethics Committee approval no. H15/03-041. Written informed consent for participation was not required for this study in accordance with the national legislation and the institutional requirements.

## Author Contributions

NG, AT-R, RB, and JA conceived the project. HF and WP collected the specimens. NG carried out the laboratory experiments and drafted the manuscript. JA supervised the project. NG, AT-R, RB, HF, WP, and JA critically reviewed and revised various versions of the manuscript. RB was co-authored this manuscript in his personal capacity and his capacity as an adjunct academic at Central Queensland University. All authors read and approved the final manuscript.

## Conflict of Interest

The authors declare that the research was conducted in the absence of any commercial or financial relationships that could be construed as a potential conflict of interest.
